# Interactions between Human Liver Fatty Acid Binding Protein and Peroxisome Proliferator Activated Receptor Selective Drugs

**DOI:** 10.1155/2013/938401

**Published:** 2013-02-18

**Authors:** Tony Velkov

**Affiliations:** Drug Delivery, Disposition and Dynamics, Monash Institute of Pharmaceutical Sciences, Monash University, Parkville Campus, 381 Royal Parade, Parkville, VIC 3052, Australia

## Abstract

Fatty acid binding proteins (FABPs) act as intracellular shuttles for fatty acids as well as lipophilic xenobiotics to the nucleus, where these ligands are released to a group of nuclear receptors called the peroxisome proliferator activated receptors (PPARs). PPAR mediated gene activation is ultimately involved in maintenance of cellular homeostasis through the transcriptional regulation of metabolic enzymes and transporters that target the activating ligand. Here we show that liver- (L-) FABP displays a high binding affinity for PPAR subtype selective drugs. NMR chemical shift perturbation mapping and proteolytic protection experiments show that the binding of the PPAR subtype selective drugs produces conformational changes that stabilize the portal region of L-FABP. NMR chemical shift perturbation studies also revealed that L-FABP can form a complex with the PPAR ligand binding domain (LBD) of PPAR**α**. This protein-protein interaction may represent a mechanism for facilitating the activation of PPAR transcriptional activity via the direct channeling of ligands between the binding pocket of L-FABP and the PPAR**α**LBD. The role of L-FABP in the delivery of ligands directly to PPAR**α** via this channeling mechanism has important implications for regulatory pathways that mediate xenobiotic responses and host protection in tissues such as the small intestine and the liver where L-FABP is highly expressed.

## 1. Introduction

Intracellular long-chain fatty acids (FAs) are key components in the synthesis of cellular membranes as well as being utilized as signaling molecules and for energy delivery [1, 2]. The preservation of a proper balance between absorption, secretion, and storage of FA is therefore, integral for cellular physiology [1]. Increasingly prominent diseases such as obesity, cardiovascular diseases, type II diabetes, and atherosclerosis, to a large extent, all evolve from disorders of lipid metabolism. *In vivo,* due to their poor aqueous solubility, FAs are bound and transported by a class of intracellular lipid binding proteins (iLBPs) termed fatty acid binding proteins (FABPs) [1–4]. Structurally, FABPs possess a similar tertiary fold, consisting of ten antiparallel *β*-strands, which form a clam shell-like *β*-barrel structure (*cf.* Figure 3(c)) [3, 5, 6]. The *β*-barrel is capped by a pair of *α*-helices that enclose an internal cavity, which forms the ligand binding pocket. A mechanism for ligand binding termed the “portal hypothesis” has been proposed, where the FA molecule enters the protein through a dynamic structure formed by the *α*-helical region, before binding inside the cavity [7–9].

The expression of genes involved in FA metabolism and glucose homeostasis is controlled by nuclear hormone receptors (NHRs), in particular a class of NHRs known as peroxisome proliferator activated receptors (PPARs) [10–12]. PPARs are ligand-activated transcription factors that respond to FA and eicosanoids [10–13]. Three isotypes of human PPAR, termed *α*, *γ*, and *δ*, have been identified each with a specific tissue distribution [12, 14, 15]. PPAR*α* and *γ* are the most studied isotypes: PPAR*α* modulates FA metabolism and glucose homeostasis in the liver and skeletal muscle, whereas PPAR*γ* modulates adipogenesis and adipocyte FA metabolism [12, 16–18]. The physiological role of PPAR*δ* is the least understood of the three human PPAR isotypes. However, not unlike the other two isotypes, PPAR*δ* binds FA and eicosanoids, signifying a regulatory role in lipid metabolism [12, 17–19]. Dysfunction of these regulatory functions of PPARs leads to the manifestation of the aforementioned human diseases. Accordingly, PPARs are important targets for antidyslipidemic drugs [16, 20, 21]. The fibrate hypolipidemic drug classes preferentially bind PPAR*α*, whereas the thiazolidinediones, which specifically bind PPAR*γ*, are used in the treatment of type II diabetes [16, 19–22]. In light of the central regulatory role of PPARs in lipid homeostasis, it follows that the development of novel therapeutic ligands with improved pharmacological profiles to target these NHRs has become an important research priority in the pharmaceutical industry [20, 21]. 

The most prevalent iLBPs in the enterocyte, the innate intestinal- (I-) FABP and L-FABP together, constitute ~3%–6% of the total cytosolic protein [23, 24]. While L-FABP is known to bind FA with high affinity, in recent reports we have shown that both L- and I-FABP can specifically bind a structurally diverse set of non-FA lipophilic drugs [25–29]. In enterocytes, all three PPAR sub-types are present, the PPAR*α* and PPAR*δ* subtypes are predominantly expressed, and to a lesser extent PPAR*γ* [12, 14, 15]. Whereas, in hepatocytes, L-FABP is highly expressed together with PPAR*α*, PPAR*δ* and PPAR*γ* are also expressed to a lesser extent [12, 14, 15, 30]. Given the high abundance of these FABPs in the intestinal epithelia, it is tenable that L- and I-FABP potentially facilitate the intestinal absorption and trafficking of lipophilic drugs to their PPAR targets.

The molecular events that underlie the transcriptional activities of PPAR selective drugs have become increasingly clear in recent years [10, 11, 16, 17, 31]. A key question that remained elusive for many years, however, is how these poorly water soluble compounds are solubilised and transported through the essentially aqueous cytosol in order to bind to their target PPAR and initiate transcription. A steadily mounting critical mass of data from several laboratories supports the notion that the nucleocytoplasmic transport of lipophilic NHR ligands is mediated by iLBPs [32–50]. 

Over a series of exemplary reports, researchers from the laboratories of Schroeder and Spener have provided convincing evidence that L-FABP directly interacts with PPAR*α* and is involved in the nucleo-cytoplasmic shuttling of activator ligands [36, 39, 41, 46, 47, 49, 50]. This study broadens this knowledge base and examines the binding characteristics of human L-FABP for PPAR sub-type selective drugs. In addition, the PPAR*α*LBD binding interface on L-FABP is mapped using NMR chemical shift perturbation experiments. The functional mechanisms inferred from the biophysical data have broad implications for current models of ligand-dependant nucleo-cytoplasmic channeling processes between FABPs and PPARs.

## 2. Materials and Methods

### 2.1. Materials

Isopropyl *β*-D-thiogalactopyranoside (IPTG) was purchased from BioVectra (Prince Edward Island, Canada). Sequencing-grade trypsin was purchased from Promega (NSW, Australia). PPAR selective drug compounds were obtained from Sigma-Aldrich (Sydney, NSW, Australia) and Cayman Chemicals (Ann Arbor, MI, USA). *Escherichia coli *strain BL21 Codon Plus (DE3)-RIL was purchased from Stratagene (La Jolla, CA, USA). ^15^NH_4_Cl was purchased from Cambridge Isotopes (Melbourne, VIC, Australia). All other reagents were of the highest purity commercially available.

### 2.2. Protein Expression and Purification

**Figure 1:**
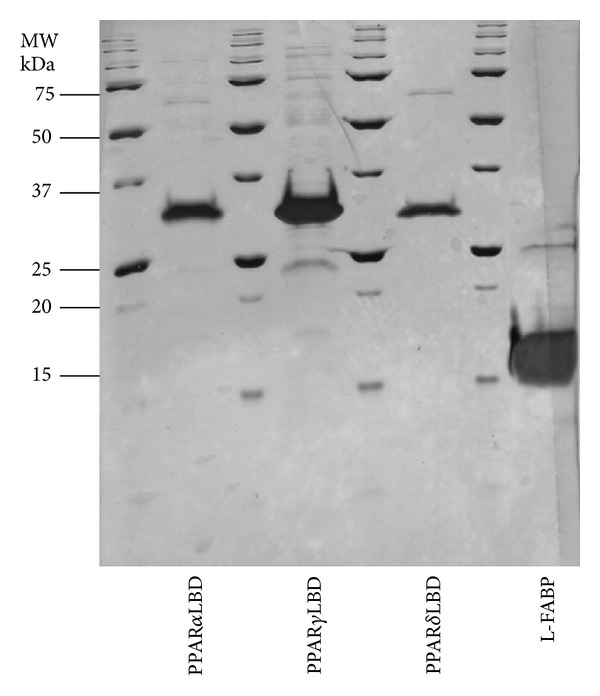
Purity of protein preparations used in this study. Protein samples were resolved by SDS-PAGE on 15% polyacrylamide gels and stained with silver. Gel lanes were loaded with ≥5 *μ*g of protein, to allow for identification of residual contaminating protein species. Molecular weight standards are indicated on the ordinate.

The expression plasmid for human L-FABP was developed internally and is available from the Plasmid Repository (http://plasmid.med.harvard.edu/PLASMID/) under the plasmid identification codes HsCD00073511. The expression plasmid for the LBD of human PPAR*α* (aa 196–468) and human PPAR*γ* (aa 193–475) was kindly supplied by Krister Bamberg, Department of Molecular Biology, Astra-Zeneca R&D Mölndol [51]. The expression plasmid for human PPAR*δ* (aa 171–441) was supplied by William Hunter, University of Dundee [52]. Following IPTG induction at a cell density of 0.6, recombinant proteins were expressed for 6 hrs and purified from *E. coli* BL21 Codon Plus (DE3)-RIL cells. ^15^N labeled L-FABP protein for NMR experiments was produced by over expression in M9 minimal media containing ^15^NH_4_Cl using the protocol of Marley et al. [53]. All proteins were engineered with N-terminal [His]_6_ affinity tags and were separated from the bulk contaminants in the soluble cell fraction by Ni^2+^-based immobilized metal ion affinity chromatography (IMAC) on Ni^2+^ Sepharose 5 mL HisTrap HP chromatography column (GE Health Care, Sydney, NSW, Australia, cat. no. 17-5248-02). Proteins were resolved using a step gradient of 0–300 mM imidazole in buffer A (50 mM Tris-HCl, pH 8.0; 500 mM NaCl; 0.5 mM ethylenediaminetetraacetic acid (EDTA); 1 mM dithiothreitol (DTT); 5% (v/v) glycerol) at a flow rate of 5 mL/min (4 column volumes (CVs) washout unbound sample; 0%–30% imidazole over 5 CVs; hold 30% for 2 CVs; 30%–100% imidazole over 5 CVs; hold 100% for 3 CVs). Delipidation and further purification was achieved by HIC on a Phenyl HP 16/10 column (GE Health Care, Sydney, NSW, Australia, cat. no. 17-1085-01) as previously described [54]. The final purity of the proteins was ascertained by SDS-PAGE (silver staining) and in all cases was >98% (Figure 1). 

### 2.3. Protein Characterization

Purified recombinant protein samples were subjected to an in-gel 16 hr tryptic digest at 37°C. The resulting peptides were extracted from the gel by zip-tip (Millipore Perfect Pure C18). A 1 *μ*L aliquot was spotted onto a sample plate with 1 *μ*L of matrix (*α*-cyano-4-hydroxycinnamic acid, 4 mg/mL in 70% (v/v) AcN, and 0.1% (v/v)) trifluoroacetic acid and allowed to air dry. Matrix-assisted laser desorption ionisation mass spectrometry was performed with an ABI 4700 time-of-flight mass spectrometer. An Nd : YAG (355 nm) was used to irradiate the sample. The spectra were acquired in reflectron mode in the mass range 750 to 3500 Da. A near point calibration was applied and will give a typical mass accuracy of ~50 ppm or less. The peptide masses were searched against *Homo sapiens *using the SWISS-PROT database with a peptide mass tolerance of 50 ppm. The analysis of the recombinant proteins indicated positive identification for human PPAR sub-types and human L-FABP.

### 2.4. NMR ^1^HN and ^15^N Backbone Amide Chemical Shift Mapping

For drug titration experiments, 600 *μ*L samples of 50 *μ*M ^15^N L-FABP were prepared in 95% H_2_O/10% D_2_O in buffer B (20 mM MES, pH 5.5; 50 mM NaCl). Drugs were titrated into the protein solution from a DMSO stock solution, and the final DMSO level was <2% (v/v). The binding surface of PPAR*α*LBD on L-FABP was mapped by titration of 50 *μ*M ^15^N-L-FABP with a nonlabelled PPAR*α*LBD (0–250 *μ*M). 2D ^1^H-^15^N HSQC spectra were acquired on a Varian ANOVA 600 MHz spectrometer operating at 20°C. Spectra were processed with the software package NMRPipe and assigned using the program SPARKY. The combined ^1^H and ^15^N backbone amide nuclei chemical shift changes between apo- and holo-L-FABP assignments were calculated using the square root of the sum of the weighted squares of the ^1^HN and ^15^N backbone amide chemical shift values (1) [55, 56] as follows:

(1) $$\begin{array}{c} \Delta \delta_{\text{comb}}=\;\sqrt{{\left(\omega_{\text{HN}}\Delta \delta^{1}\text{H}\right)}^{2}+{\left(\omega_{\text{N}}\Delta \delta^{15}\text{N}\right)}^{2}}, \end{array}$$

where Δ*δ* ^1^HN and Δ*δ* ^15^N denote the ^1^HN and ^15^N backbone amide chemical shift change between the apo*-* and holoprotein for a particular residue, and *ω*_*i*_ denotes the weight factor of the nucleus which accounts for differences in sensitivity of the ^1^HN and ^15^N *ω*_NH_ = 1.0; *ω*_NH_ = 0.154. Weight factors are determined from the ratio of the average standard deviations of the chemical shifts for a given nucleus type observed for the 20 proteogenic amino acids using the BioMagResBank chemical shift database [57]. The Δ*δ*_comb_ for each titration was normalized to the maximum Δ*δ*_comb_ for the given data set. Residues that displayed chemical shift perturbations >0.6 p.p.m were mapped onto the crystal structure of human L-FABP (PDB code: 2F73) to visualize the movement of backbone amides. Molecular visualizations were performed using the software packages PYMOL (Delano Scientific, San Carlos, CA, USA).

### 2.5. Ligand Binding Fluorescence Measurements

Fluorometric protein-ligand binding affinity measurements were performed under steady-state conditions on a Cary Eclipse fluorescence spectrophotometer (Varian, Mulgrave, VIC, Australia) in buffer C (20 mM Tris-HCl, pH 7.4; 50 mM NaCl; 0.5 mM EDTA; 1 mM DTT; 5% (v/v) glycerol) at 20°C. The binding assay buffer for L-FABP did not contain glycerol, and all other components were identical. The drug binding affinity of L-FABP was measured fluorometrically by monitoring the displacement of the fluorescent binding cavity probe 1-anilino-8-naphthalenesulfonic acid (ANS) as previously described [28, 58]. The binding properties of PPAR LBDs and L-FABP were measured by monitoring the displacement of the fluorescent FA *cis*-parinaric acid [59]. Competition experiments were performed where PPAR LBDs (1 *μ*M) preincubated with *cis*-parinaric acid (2 *μ*M) were titrated with a competing ligand. The decrease in fluorescence upon addition of competing ligand was monitored and plotted as a function of the concentration of free ligand. Displacement data were fitted by nonlinear regression to a one-site or where indicated two-site competition models from which inhibition constant (*K*_*i*_) values were derived [25, 28, 58]. All data modeling operations were performed using GraphPad Prism V5.0 software (GraphPad software, San Diego, CA, USA).

### 2.6. Limited Proteolysis of L-FABP

 The limited proteolysis of L-FABP (50 *μ*M) with sequencing grade trypsin was carried out in buffer D (20 mM Tris-HCl, pH 8.0; 50 mM NaCl; 2 mM CaCl_2_) for 1 hr at 20°C. All digestions were performed at a protein:protease ratio of 20 : 1 (w/w). Apo- or holo-drug (0, 0.1, 0.4, 1.0, 5 *μ*M final drug concentration) protein samples were equilibrated with the drug at 20°C for 15 min before protease was added. Digestion reactions were stopped by the addition of 5 *μ*L of 50 mM phenylmethanesulfonyl fluoride (PMSF) followed by one volume of SDS-PAGE sample buffer (12.5% 0.5 M Tris-HCl pH 6.8; 0.005% bromophenol blue; 10% SDS; 10% glycerol; 2%  *β*-mercaptoethanol) and heated for 2 min at 100°C. Samples were resolved on 4% stacking, 20% resolving polyacrylamide gels at 4°C at a constant voltage of 80 V, using the Laemmli buffer system. Gels were stained with Coomassie Blue G-250 and destained with 50% methanol/10% acetic acid (v/v) solution. Gels were dried between cellulose sheets and scanned at 1200 dpi. Protein bands were quantified densitometrically using LabImage 1D gel analysis software V3.4 (Kapelan GmbH, http://www.kapelan-bioimaging.com/). 

### 2.7. PPAR*α*LBD Protection from Limited Proteolysis through Interactions with L-FABP

Protein mixtures of PPAR*α*LBD (30 *μ*M) and L-FABP (0, 50, 100 *μ*M) were digested with a final protease concentration of 2 *μ*M in digestion buffer D for 30 min at 20°C. In some reactions, PPAR*α*LBD (30 *μ*M) was preequilibrated for 30 min with varying concentrations of GW7647 (0, 1, 5 *μ*M) prior to digestion. The reaction was quenched by the addition of PMSF and one volume of SDS-PAGE sample buffer as detailed above. Samples were resolved by 15% resolving SDS-PAGE polyacrylamide gels then transferred to a PVDF membrane and probed with anti-human PPAR*α* antibody (mapping to C-terminal aa 420–468 of the PPAR*α*LBD; cat. no. sc1982 Santa-Cruz Biotechnology, Santa Cruz, CA, USA) by western blotting.

### 2.8. Differential Scanning Calorimetry (DSC)

DSC measurements were performed on N-DSC II calorimeter (Calorimetry Sciences Corporation, UT, USA) at a heating rate of 0.5°C/min. For preparation of 250 *μ*M protein sample solutions, L-FABP was extensively buffer exchanged into buffer E (20 mM Tris-HCl pH 8.0) by ultrafiltration using Amicon Ultra 3 K centrifugal concentrators and degassed prior to filling the calorimeter cells. The filtrate from the sample preparation was used as a reference buffer for the DSC measurements. The thermal transition mid-point temperatures (*T*_*m*_) were calculated using CpCalc software (Calorimetry Sciences Corporation, UT, USA). 

## 3. Results

### 3.1. Examination of the Binding Affinity of PPAR Subtype Specific Drugs for L-FABP and PPAR LBDs by Fluorescence Displacement Assays

The binding dissociation constants (*K*_*i*_) of PPAR sub-type selective drugs were measured fluorometrically by monitoring the competitive displacement of the fluorescent probes, ANS and *cis*-parinaric acid from the ligand binding cavity of L-FABP or PPAR LBDs, respectively (Table 1). The binding affinity of the nonfluorescent ligand is determined from the EC50 of the competition curve and the *K*_d_ of the fluorescent probe [28, 58]. Binding isotherms for L-FABP conformed well to a one-site binding model [28, 58], whereas the model did not converge with attempts to fit a two-site model [25]. The test compounds are all highly lipophilic; the apparent octanol-water partition coefficient for each drug at pH7.4 (log*D*_7.4_) is documented in Table 1 as a measure of their lipophilicity. The rank order of affinity from the highest to the lowest L-FABP binding affinity was GW7647 > fenofibric acid > L165,041 ≥ GW1929 ≥ troglitazone ≥ Rosiglitazone > GW590735 (Table 1). There does not appear to be any direct correlation between the L-FABP drug binding affinity and the log*P* or log*D*_7.4_ of the compounds, which implies that the interactions are more likely driven by the molecular specificity of the binding cavity. In order to probe if the drugs bind to both fatty acid binding sites in the L-FABP cavity, displacement titrations for GW7647 and L165,041 were performed using *cis*-parinaric acid, a fluorescent probe which has been previously shown to occupy both cavity sites [60–62]. Oleate was used as a control ligand and displayed the expected 2-site binding behavior (Table 1). Comparably, the GW7647 and L165,041 displacement data did not fit to a two-site competition model and only conformed to the one-site competition model, which yielded *K*_*i*_ values similar to the ANS displacement assay. Moreover, the drugs could not completely displace the *cis*-parinaric acid fluorescence, suggesting that they only bind to one of the cavity sites.

The PPAR LBDs displayed the expected high selectivity for their respective sub-type selective ligands (Table 1). Generally, PPAR LBD ligand sub-type selectivity is determined by the polar head group (carboxylic acid or thiazolidinedione ring) making precise hydrogen bonding interactions within the binding pocket, and the rest of the ligand is well tolerated. PPAR*γ* and *α* have larger binding pockets compared to PPAR*δ*, which has a narrower pocket where the polar head group is accommodated [20, 21, 52]. The major determinant of ligand selectivity between the PPAR*γ* and *α* subtypes is the substitution of Tyr^314^ in PPAR*α* for His^323^ in PPAR*γ* [21]. These amino acids form part of the hydrogen bonding network involved in stabilizing the polar head group of the ligand. The larger volume of Tyr^314^ prevents ligands with large substituents proximal to their head group from being accommodated properly. For example, the large benzophenone group proximal to the head group of GW1929 results in a ~48-fold greater selectivity for PPAR*γ* over PPAR*α* (Table 1). The pocket that accommodates the head group is significantly smaller in PPAR*δ*; thus thiazolidinediones and ligands with large substituents near their head groups do not avidly bind to PPAR*δ*. The potent PPAR*δ* agonist L165,041 contains an unsubstituted phenoxyacetic acid head group that can fit into the narrow binding pocket. Similarly, the small alkyl substituents adjacent to the carboxylate group of fenofibric acid allow it to bind PPAR*δ* with a moderate binding affinity (Table 1). 

### 3.2. Conformational Changes in L-FABP Induced by PPAR Selective Drug Binding Monitored by ^1^H and ^15^N Backbone Amide Chemical Shift Mapping

**Figure 2:**
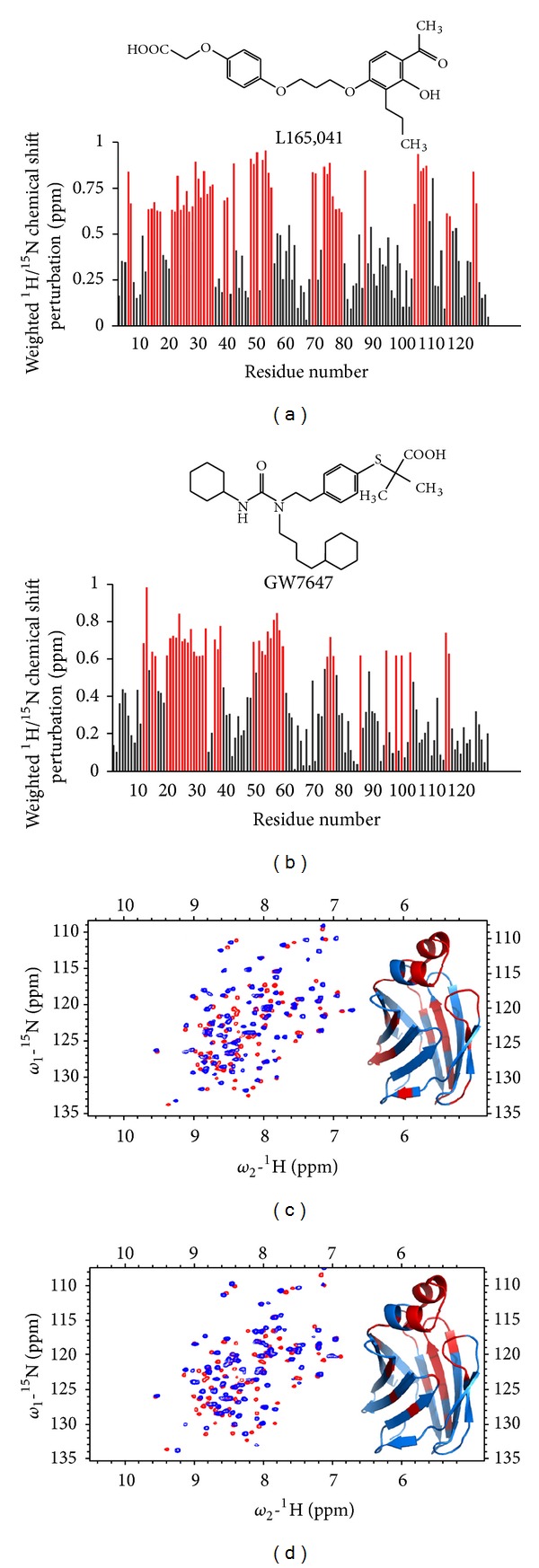
^1^HN and ^15^N backbone amide chemical shift perturbations on human L-FABP produced by GW7647 and L165,041 drug binding. ((a)-(b)) A plot of the normalized chemical shift perturbations upon complex formation versus residue number. Red bars indicate highly affected residues (>0.6 p.p.m perturbation). ((c)-(d)) ^1^H-^15^N HSQC spectrum of apo-L-FABP (red) overlaid on top of the spectrum of holo-drug L-FABP (blue).The inset shows the highly affected residues (>0.6 ppm perturbation) mapped onto the crystallographic structure of human L-FABP (PDB code: 2F73).

Local conformational changes induced by ligand binding can be monitored by ^1^HN and ^15^N backbone amide chemical shift changes that are related to the change of the dihedral *ϕ*, *ψ*-angles [56]. Changes in chemical shift were followed by recording a series of ^1^H-^15^N-HSQC spectra of L-FABP in the presence of increasing concentrations of PPAR*α* and PPAR*δ* sub-type selective drugs GW7647 and L165,041, respectively (Figure 2). Mapping of the chemical shift perturbations between the apo- and holo-drug-L-FABP complexes onto the three-dimensional crystal structure of human L-FABP (PDB code: 2F73) revealed that the most significant perturbations were concentrated within the binding cavity or the portal region that mediates ligand entry/exit from the *β*-barrel cavity (Figure 2). 

### 3.3. Binding to PPAR Selective Drugs Protects L-FABP from Limited Proteolysis and Heat Denaturation

**Figure 3:**
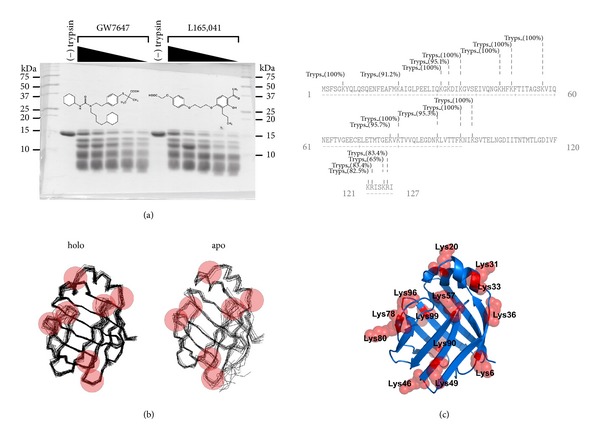
Binding to PPAR selective drugs protects L-FABP from limited proteolysis. (a) L-FABP was preequilibrated with increasing concentrations (0, 0.1, 0.4, 1.0, 5 *μ*M) of GW7647 or L165,041 and then digested with trypsin. The resultant fragments were resolved on 20% polyacrylamide gels and visualized by Coomassie Blue G250 staining. Molecular weight standards are indicated on the ordinate. (b) The red spheres highlight the predicted tryptic cleavage hot spots on backbone overlays of the ensemble of structures determined by solution-state NMR for apo- (PDB code: 2PY1) and holo- (PDB code: 2L68) human L-FABP. (c) Top panel, tryptic cleavage sites predicted by the ExPASy PeptideCutter algorithm mapped onto the amino acid sequence of human L-FABP. The probability of cleavage at each site is indicated in parentheses. Bottom panel, the highest scoring sites (>90% probability) are mapped onto the crystallographic structure of human L-FABP (PDB code: 2F73).

Limited proteolysis is a useful method for examining the location of ligand binding sites or associated conformational changes from the observed differential susceptibility of proteolytic sites. We have examined the proteolytic peptide pattern that evolves from the digestion of L-FABP with trypsin (Arg-C; Lys-C) in the apo- and holo-GW7647 and L165,041 drug bound forms (Figure 3(a)). The apoform was significantly more susceptible to proteolysis than the holo-GW7647 and L165,041 drug bound forms. The relative proteolytic susceptibility of each drug-L-FABP complex was proportional to the binding affinity of the ligand, such that GW7647 which has a higher L-FABP binding affinity afforded more resistance to proteolysis. The dependence of proteolytic susceptibility on the binding affinity of each ligand could be demonstrated in a concentration-dependant manner. Control experiments with *E.coli* lac repressor, a protein that does not possess an affinity for the test compounds, showed no protection against cleavage, thus ruling out the possibility that the protection observed with L-FABP is due to an inhibitory effect from each ligand on the protease (data not shown). Potential tryptic cleavage sites were predicted using the ExPASy PeptideCutter algorithm (http://web.expasy.org/peptide_cutter/) and mapped onto the crystallographic structure of human L-FABP (Figures 3(b) and 3(c)). The highest scoring Lys residues are situated within the portal helices and the loop regions between adjoining *β*-sheets (Figures 3(b) and 3(c)). 

**Figure 4:**
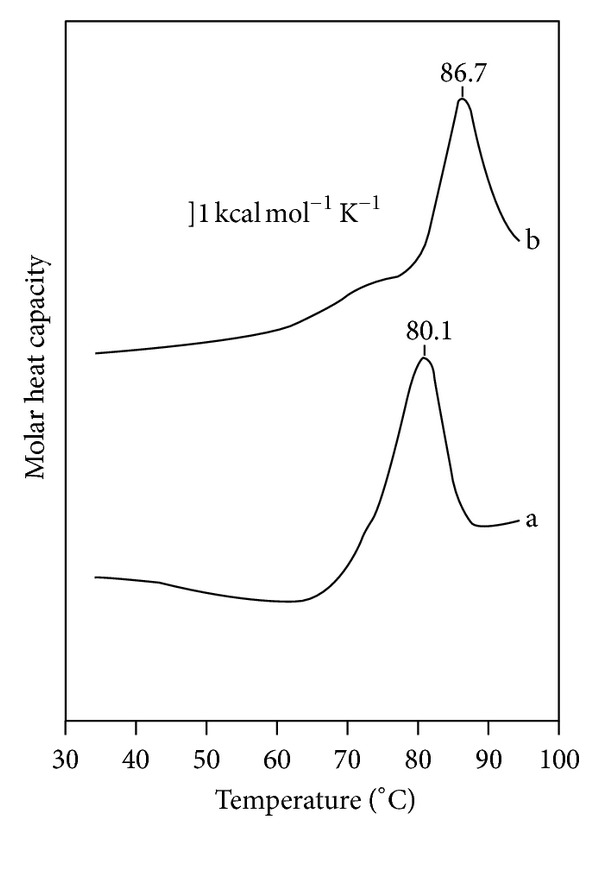
DSC thermograms of (a) apo- and (b) holo-GW7647 human L-FABP. The *T*_*m*_ is indicated above each thermogram. The scan rate was 0.5°C/min.

DSC thermal denaturation measurements reveled complexation with GW7647 increased the thermal transition mid-point temperature of L-FABP from *T*_*m*_ = 80.1 to *T*_*m*_ = 86.7 (Figure 4), which would indicate that drug binding stabilizes the L-FABP structure.

### 3.4. Investigation of L-FABP-PPAR*α*LBD Protein-Protein Interactions

#### 3.4.1. ^1^HN and ^15^N Backbone Amide Chemical Shift Mapping of the PPAR*α* LBD Binding Surface on L-FABP

**Figure 5:**
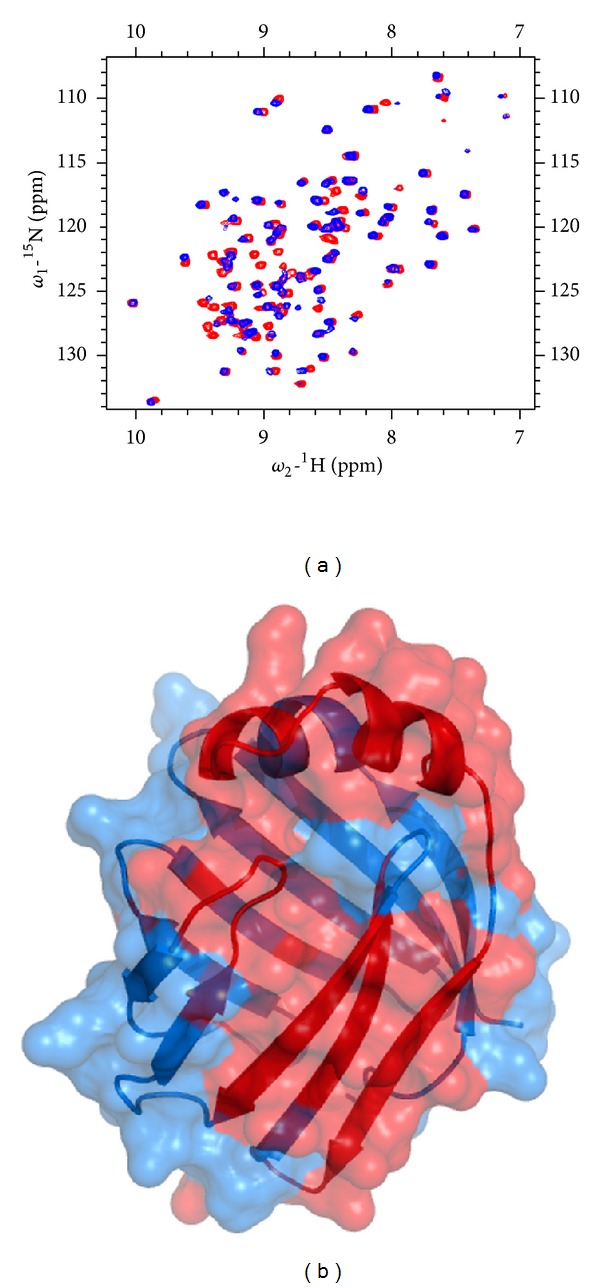
(a) ^1^H-^15^N HSQC spectrum of apo-L-FABP (red) overlaid on top of the spectrum of holo-PPAR*α*LBD-L-FABP (blue). (b) The highly affected residues (>0.6 p.p.m perturbation) are mapped onto the crystallographic structure of human L-FABP (PDB code: 2F73).

To investigate the ability of the PPAR*α*LBD to interact directly with L-FABP and map the protein-protein interaction surface, ^1^HN-^15^N HSQC spectra of ^15^N-labelled L-FABP were acquired in the presence of nonlabeled PPAR*α*LBD (Figure 5). Titration of ^15^N-L-FABP with PPAR*α*LBD produced a perturbation of a number of resonances (Figure 5(a)). Mapping the chemical shift perturbations onto the three-dimensional structure of L-FABP indicated that PPAR*α* LBD binding predominantly affects surface residues situated in the portal region, within the *β*E-*β*F loop segment, *β*B-*β*D sheets, and residues localized at the bottom of the binding cavity (Figure 5(b)). 

#### 3.4.2. PPAR*α*LBD Protection from Limited Proteolysis at the L-FABP Protein-Protein Interaction Interface

**Figure 6:**
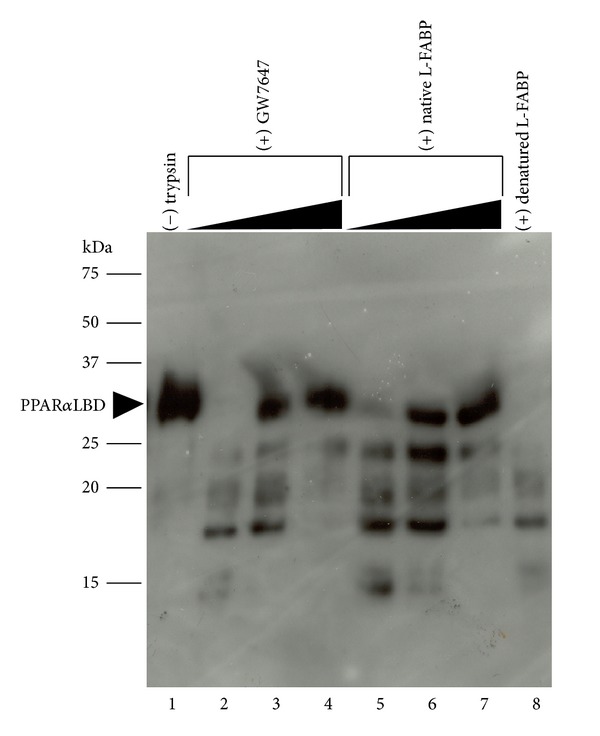
Proteolytic protection of PPAR*α*LBD through interactions with L-FABP. A fixed concentration of PPAR*α*LBD (30 *μ*M) was digested with trypsin in the presence of increasing concentrations of native LFABP (0, 50, 100 *μ*M). Control protein digests were performed in the presence of increasing concentrations of the PPAR*α* selective drug GW7647 (0, 1, 5 *μ*M) or heat denatured L-FABP. The gel was probed by western blotting with a PPAR*α*LBD antibody. The position of the intact PPAR*α* LBD protein band is indicated.

A series of partial proteolysis reactions were performed where a fixed concentration of PPAR*α*LBD (30 *μ*M) was digested in the presence of increasing concentrations of L-FABP (0, 50, 100 *μ*M) (Figure 6). The gel was probed for PPAR*α*LBD fragments by western blotting with a PPAR*α*LBD C-terminal specific anti-body (aa 420–468). PPAR*α*LBD was more resistant to proteolysis in the presence of L-FABP compared to digestions of PPAR*α*LBD protein *per se *(Figure 6). In order to demonstrate that the proteolytic protection of PPAR*α*LBD is not due to the sequestration of protease from the preferential cleavage of L-FABP, the same experiments were performed with heat denatured L-FABP, which afforded very little protection of PPAR*α*LBD (Figure 6). The binding of the PPAR*α*LBD selective drug GW7647 also afforded protection to the PPAR*α*LBD against proteolysis (Figure 6). Taken together, these findings demonstrate that the complexation of the PPAR*α*LBD with either L-FABP or a high affinity ligand stabilizes its structure, making it more resilient to proteolytic attack.

## 4. Discussion

Our understanding of how ligand binding events to specific NHRs produce a biological response on the activating ligand is mostly limited to transcriptional events in the nucleus, whereas mechanisms of selective accumulation and intracellular transport of ligands to their selective NHR remain largely unknown. The pivotal role played by iLBPs in xenobiotic mediated transactivation of NHRs is only beginning to become apparent. Several groups have reported a direct link between the regulation of NHR transcriptional activity through iLBP mediated ligand delivery to the nucleus [32–50, 63]. It is becoming increasingly apparent that tissue specific FABPs act as solubilizing intracellular shuttles for lipophilic ligands to the nucleus, where the ligand is released to its target PPAR. In a broader cellular context, this nucleo-cytoplasmic shuttling mechanism is believed to be one component of a larger feedback loop for both endogenous and exogenous lipophilic ligands to the nucleus, where NHR activation modulates the expression of metabolic enzymes and transporters such as L-FABP that help detoxify the ligand [37, 45–48]. The current study aims to provide additional insight into this feedback mechanism, in particular, the role of L-FABP in binding PPAR selective drugs and promoting interactions with PPAR LBDs.

The inhibitory binding affinity constants (*K*_*i*_) characterizing the interaction of human L-FABP with a set of PPAR sub-type selective lipophilic drugs were measured by fluorescence competition assays (Table 1). L-FABP displayed a broad selectivity with a high binding affinity for most of the compounds tested. Binding isotherms for L-FABP only conformed well to a one-site binding model [28, 58]. This contrasts the two oleate molecules bound per L-FABP monomer in the crystallographic and NMR structures [64–67], which is also coincident with the *cis*-parinaric acid displacement data for oleate (Table 1). Presumably, the elaborate structures of these drugs only allow for accommodation of one drug in the binding cavity. However, it should be noted that one limitation of the fluorometric assay platform is that it measures the displacement of ANS, a probe which only binds to one of the two ligand sites in the cavity of L-FABP. Nevertheless, the one-site binding behavior of the drugs was also evident with displacement titrations using *cis*-parinaric acid, the fluorescent probe which is known to occupy both cavity sites [60–62]. The overlapping ligand selectivity of L-FABP and the PPAR LBDs suggests that they may cooperate in the transduction of the biological activities of their shared ligands. The high affinity of the PPAR selective drugs for L-FABP can be potentially attributed to their polar head groups (carboxylic acid or thiazolidinedione ring) which likely mimic the interactions involving the FA head group seen in the crystallographic complex [65, 66]. In most cases, L-FABP binds these compounds with an affinity about an order of magnitude lower than their selective PPAR LBD. This possibly reflects the situation *in vivo* where once L-FABP has entered the nucleus, the affinity gradient would promote transfer of the ligand to the PPAR LBD. 

**Figure 7:**
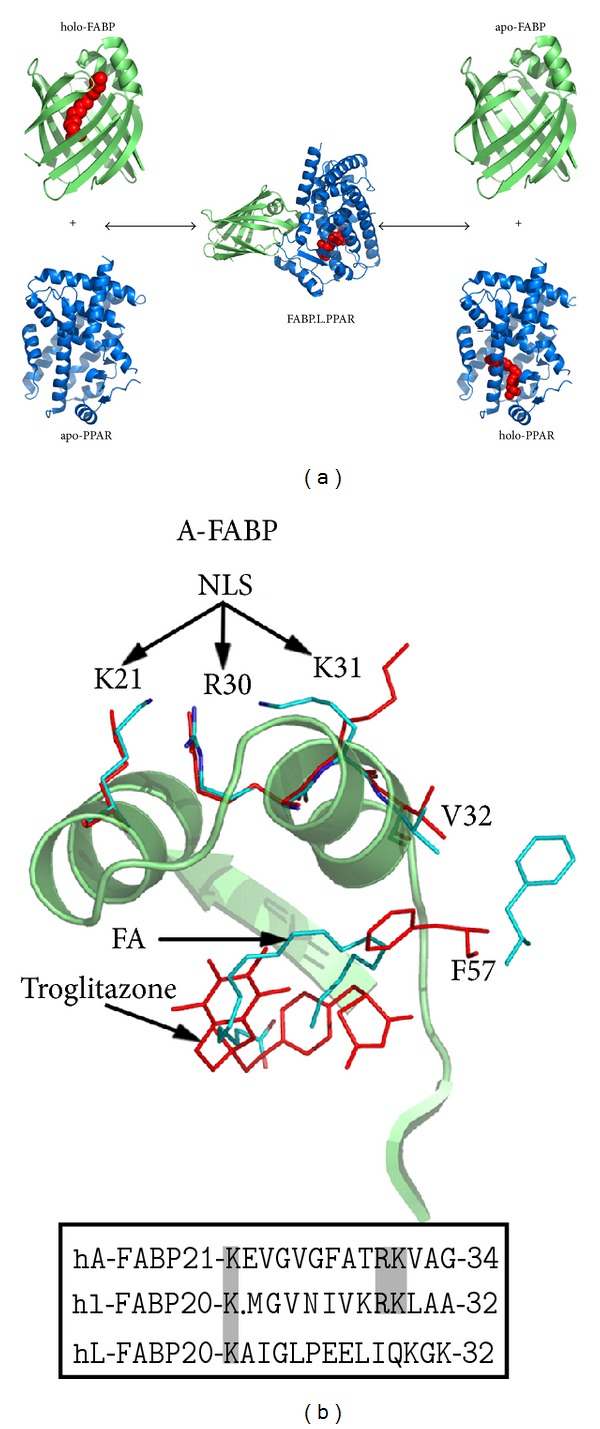
(a) The conformational nuclear localisation signal formed in A-FABP upon binding of an activating ligand. The portal region of A-FABP is shown in ribbon representation in complex with an activator ligand troglitazone (red; PDB code: 2QM9.pdb) and in complex with a non-activator ligand, arachidonic acid (cyan; PDB code: 1ADL.pdb). The side chains of the NLS residues (Lys^21^, Arg^30^, Lys^31^) and cavity lid residue (Phe^57^) are shown in sticks representation. Below, sequence alignment of the NLS region of human A-FABP, with human I- and L-FABP. NLS residues are highlighted in bold on a grey background. (b) Putative mechanism for the transfer of ligands from FABPs to PPAR LBDs.

The internalized ligand binding cavity of FABPs means the bound ligand is inaccessible to the aqueous milieu. This property allows FABPs to solubilize and shield relatively insoluble and potentially toxic ligands. However, internalization of the ligand presents the problem as to how PPARs that need to recognize its presence gain access. The direct transfer of the ligand through a transient protein-protein interaction with the ligand binding domain of the cognate PPAR would solve these issues. Chemical shift perturbation experiments demonstrated that L-FABP can directly interact with the PPAR*α*LBD (Figure 5). The main structural elements of L-FABP that interact with the PPAR*α*LBD are the portal region, *β*B-*β*D sheets, and the hairpin loop between the *β*E-*β*F sheets (Figure 5(b)). Considering that the protein-protein interaction interface occurs largely at the ligand binding entrance of L-FABP, this provides a direct pathway for channeling of the ligand from the *β*-barrel cavity of L-FABP into the binding pocket of the PPAR*α*LBD (Figure 7(a)). Similarly, transient protein-protein interactions have been documented for other iLBP-NHR combinations [35, 38, 40, 43–45]. Channeling of lipophilic ligands via formation of a transient FABP-PPAR complex is advantageous as it by-passes the aqueous phase, thereby the ligand remains solubilized while the interprotein exchange takes place (Figure 7(a)). Furthermore, this mechanism would allow for precise ligand targeting to the binding pocket of a sub-type specific acceptor PPAR, avoiding unwanted activation of other NHRs. However, the ability of L-FABP to avidly bind drugs selective for all three PPAR sub-types begs the question if L-FABP is involved in ligand transport to all three PPAR sub-types as opposed to specifically targeting PPAR*α*. 

The binding of PPAR activator drugs to L-FABP produces a stabilizing conformational change which has been demonstrated by limited proteolysis and DSC (Figures 3 and 4). The holo-drug L-FABP form exhibited a differential susceptibility to proteolysis that is coincident with the binding affinity of the drug such that the higher affinity drug, GW7647, afforded a greater level of protection against cleavage compared to the lower affinity compound, L165,041 (Figure 3). Chemical shift perturbation mapping data demonstrated that drug binding produces changes predominately in the portal region (Figure 2). Coincidently, a comparison of the solution structures of apo- and holo-rat L-FABP showed noticeable differences, particularly in the portal region [64]. In the rat apo-L-FABP structure, the *α*-helical region is in a more open position, presumably to allow for ligand entry into the binding cavity [64]. In contrast, a comparison of the solution structures of the apo- and holo-human L-FABP did not reveal large differences between the two forms (Figure 3(b)) [67]. As an alternative, it has been proposed that ligand binding by human L-FABP involves changes in backbone dynamics, such that a high degree of backbone flexibility in the portal region allows for ligand entry and exit from the cavity [67]. NMR backbone dynamics data demonstrated that ligand binding produces an overall stabilization of the human L-FABP structure, particularly within the *α*-helix II [67]. Thus, the higher degree of backbone flexibility would make the predicted tryptic cleavage sites in the portal region more accessible to proteolytic attack, which would account for the increased susceptibility of the apo form. The functional importance of the ligand-induced conformational changes within the portal was demonstrated in the case of A-FABP. The binding of an activator ligand to A-FABP induces conformational changes within the portal region that leads to the formation of a nuclear localization signal (NLS) (Figure 7(b)) [43, 44]. FABPs have an average molecular mass of around 15 kDa, which would allow them to freely diffuse into the nucleus through the nuclear pores. However, in adipocytes, which express A-FABP, experiments with cell-based systems demonstrated that the nuclear translocation of A-FABP is significantly enhanced following treatment with PPAR*γ* activator ligands, suggesting a controlled nuclear exclusion mechanism [43]. This represents an elegant mechanism for controlling ligand flux to NHRs. A crystallographic study of A-FABP in complex with activator and nonactivator ligands revealed the structural basis for the A-FABP-ligand interactions that induce nuclear translocation [44]. In the primary sequence of A-FABP, there are no identifiable nuclear import or export signals; however, functional forms of both motifs have been shown to exist in the three-dimensional structure of the folded protein (Figure 7(b)) [44]. Although A-FABP is capable of binding multiple ligands, only PPAR*γ* activator ligands that induce nuclear import in the cell-based systems were able to stabilize a conformational NLS situated in the *α*-helix II of the portal consisting of Lys^21^, Arg^30^, and Lys^31^ (Figure 7(b)) [44]. For example, in complex with troglitazone, a PPAR*γ* activator that induces nuclear translocation of A-FABP, the helical region of the portal is stabilized in a conformation wherein a functional NLS is exposed (Figure 7(b)). It is then believed that the activated ligand-A-FABP complex interacts with nuclear importins and the holo-A-FABP is translocated into the nucleus. Whereas, in the apo form the portal region moves freely from the *β*-barrel structure, such that the recognition site is no longer intact and therefore the apo form is not recognized by importins. Similarly, in complex with a nonactivating ligand, arachidonic acid, the conformation of the NLS residues is not stabilized and a functional NLS is not formed (Figure 7(b)). The occurrence of identical sequence elements in the portal region of I-FABP (Lys^20^, Arg^28^, Lys^29^) suggests that a similar conformational NLS may evolve in response to activator ligand binding (Figure 7(b)). Interestingly, the NLS sequence is not present in L-FABP, and this suggests that L-FABP is targeted to the nucleus via different mechanisms or that L-FABP uses a different subset of positively charged residues to form the NLS. 

### 4.1. Significance

Our findings suggest that the overlapping binding specificity between L-FABP and PPAR LBDs represents an important component in a channeling mechanism that mediates cellular transport and selective accumulation of PPAR selective drugs in the intestine and liver where both proteins are highly expressed. L-FABP may act as a gate-keeper that is responsible for communicating the state of cellular metabolism and disposition of these ligands from the cytosol to the nucleus. 

## Figures and Tables

**Table 1:** Drug binding affinity constants for human L-FABP and PPAR LBDs determined by fluorometric displacement assays.

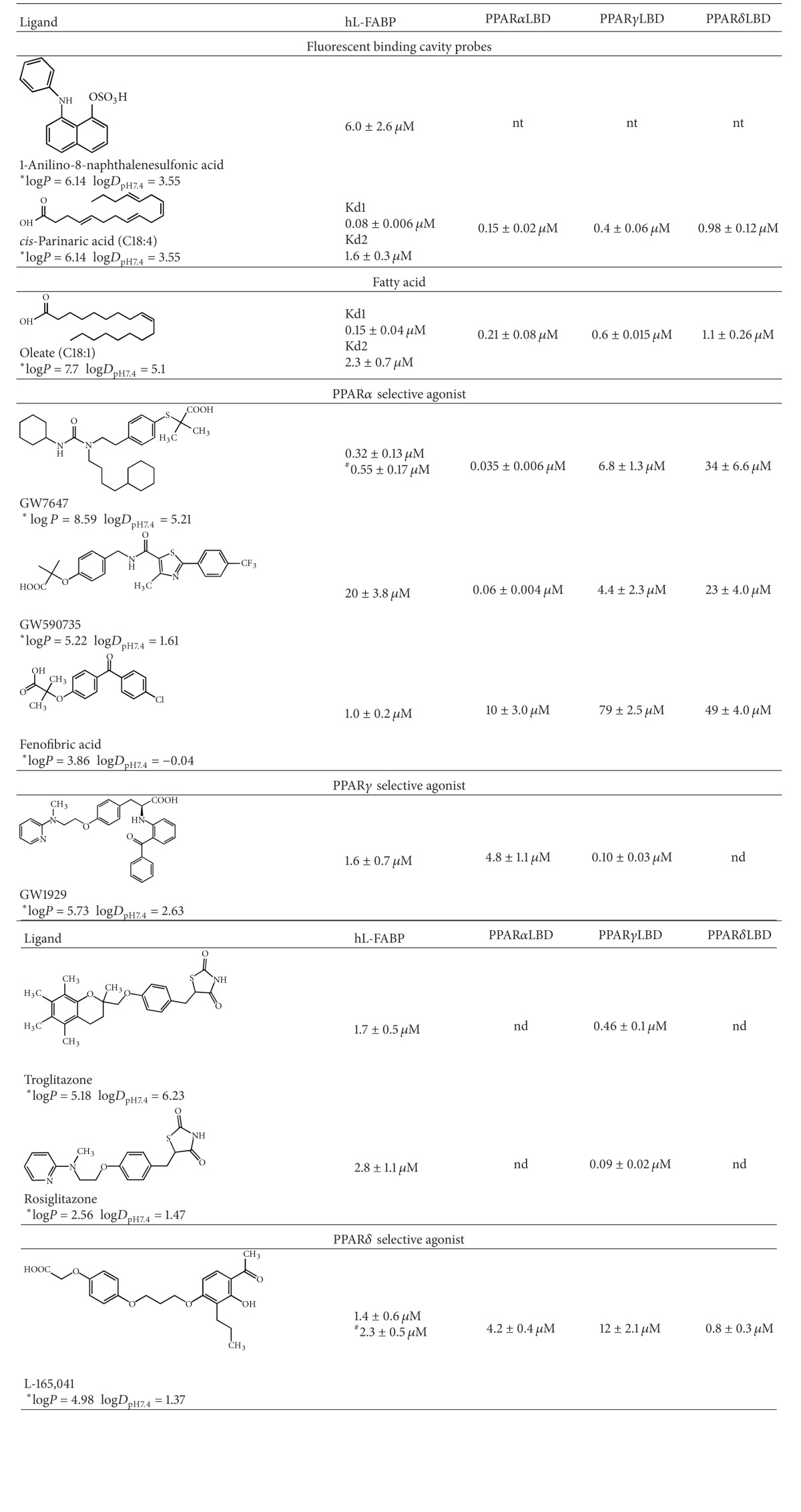

nd: no binding detected. An accurate measure of the *K*_*i*_ could not be determined due to the combination of a low binding affinity of the receptor and the poor solubility of the drug.

nt: not tested.

*The log*P* and log⁡*D*_pH7.4_ values for the compounds were calculated using the ACD Labs software.

^#^Determined by *cis*-parinaric acid displacement assay with L-FABP.

## Abbreviations

ANS: 1-Anilino-8-naphthalene sulfonic acid
FA: Long-chain fatty acid
FABP: Fatty acid binding protein
iLBP: Intracellular lipid binding protein
2D-^1^H-^15^N-HSQC: Two-dimensional amide proton and nitrogen heteronuclear single-quantum correlation
NHR: Nuclear hormone receptor.
